# Post-Marketing Safety Surveillance of Autoimmune Diseases Following Bivalent Human Papillomavirus Vaccination in China

**DOI:** 10.3390/vaccines14080687

**Published:** 2026-08-10

**Authors:** Xueyang Zeng, Xiaoshan Yu, Qiufen Zhang, Biao Rong, Moliang Chen, Huanyang Qi, Xulian Cai, Tingting Qiu, Yang Feng, Shoujie Huang, Huirong Pan, Lishan Ye

**Affiliations:** 1 Xiamen Innovax Biotech Co., Ltd., Xiamen 361022, China; xueyang.zeng@innovax.cn (X.Z.); xiaoshan.yu@innovax.cn (X.Y.); qiufen_zhang@innovax.cn (Q.Z.); tingting.qiu@innovax.cn (T.Q.); 2 Xiamen Health and Medical Big Data Center (Xiamen Medicine Research Institute), Xiamen 361008, China; 13328777315@163.com (B.R.); chengmolia@126.com (M.C.); huanyang7@163.com (H.Q.); alian08@163.com (X.C.); 3 Xiamen Chazen Biomedical Technology Co., Ltd., Xiamen 361101, China; yangfeng@chazenbio.com; 4 The State Key Laboratory of Molecular Vaccinology and Molecular Diagnostics, National Institute of Diagnostics and Vaccine Development in Infectious Diseases, School of Public Health, Xiamen University, Xiamen 361102, China; huangshoujie@xmu.edu.cn; 5 Department of Automation, Tsinghua University, Beijing 100084, China

**Keywords:** autoimmune diseases, human papillomavirus vaccine, post-marketing, real-world, safety

## Abstract

Background/Objectives: The potential of vaccines to trigger autoimmune diseases (ADs) has been extensively investigated and remains a routine focus of vaccine-safety research. This post-marketing, real-world study compared the risk of ADs between females who received bivalent human papillomavirus (HPV) vaccine (Cecolin) and unvaccinated females. Methods: Eligible females aged 9–45 years registered in the Xiamen Health and Medical Big Data Center from September 2020 to December 2023 (post-Cecolin period) were enrolled. Cecolin recipients constituted the exposed cohort, and a matched unexposed cohort was generated in a 1:4 ratio based on age and calendar year. Case validation was conducted to determine optimal identification algorithms. Incidence rates (IRs) were calculated and incidence rate ratios (IRRs) with 95% confidence intervals (CIs) were derived from zero-inflated Poisson regression. Results: Overall incidence of ADs was 70.63 per 100,000 person-years (95% CI: 67.59–73.77) in the post-Cecolin period. Compared with matched unexposed cohorts, vaccinated females showed a significantly lower AD risk in both contemporaneously matched (IRR = 0.23, 95% CI: 0.08–0.65; *p* = 0.006) and historically matched (IRR = 0.21, 95% CI: 0.07–0.66; *p* = 0.008) analyses. Conclusions: Consistent with prior evidence, this study provides real-world evidence further confirming that Cecolin vaccination does not increase the risk of ADs. It adds the first large-scale post-marketing safety evidence for this Chinese domestic bivalent HPV vaccine, filling a critical evidence gap. These results may inform vaccination policy and guide post-licensure safety monitoring in China and other countries that have introduced this vaccine.

## 1. Introduction

Extensive research has been conducted to investigate the potential association between human papillomavirus (HPV) vaccination and risk of autoimmune diseases (ADs), which continues to be a central focus of vaccine safety studies [1,2]. Several mechanisms have been proposed as possible contributors, including molecular mimicry, bystander activation, polyclonal activation, antigen diffusion, etc. [1,3,4,5]. However, the pathogenic pathways that would establish a causal link between vaccination and ADs are incompletely understood and difficult to study. HPV vaccines contain adjuvants designed to enhance immunogenicity, and these adjuvants have been theorized to trigger autoimmunity, although their precise role in the onset of ADs has not been substantiated [1,5,6]. Numerous post-marketing studies have examined the association between HPV vaccination and ADs; the overwhelming majority conclude that the vaccine does not increase the risk of ADs, yet a few discordant findings persist [7,8,9,10,11]. These anomalous signals are generally attributed to methodological artifacts—including age-related differences in the study populations, residual confounding by health-seeking behavior, inconsistent case ascertainment, heterogeneous clinical definitions, divergent study designs, or chance findings—rather than to a genuine biological effect [8,9,10,11]. These factors underscore the need for large, well-controlled post-marketing studies with rigorous case validation.

HPV vaccination is a primary prophylactic strategy against cervical cancer. Spurred by the World Health Organization (WHO) call for elimination of cervical cancer, HPV vaccine uptake has expanded rapidly, and an increasing number of countries have introduced the vaccine into their national immunization programs [12,13]. A key contributor to this expansion is the Chinese-manufactured bivalent HPV vaccine, Recombinant Human Papillomavirus Bivalent (Types 16, 18) Vaccine (*Escherichia coli*), trade-named Cecolin (Xiamen Innovax Biotech Co., Ltd., Xiamen, China). Cecolin shares the fundamental recombinant L1 virus-like particle design of other globally licensed HPV vaccines but is distinguished from the quadrivalent Gardasil and nonavalent Gardasil-9 (Merck) and the bivalent Cervarix (GlaxoSmithKline) by its *E. coli*–based expression system and aluminum hydroxide adjuvant. Under the same immunologic mechanism of HPV vaccines, Cecolin has demonstrated adequate safety and efficacy profiles, receiving marketing authorization in China in December 2019 and WHO prequalification in October 2021 [14,15]. As of September 2025, Cecolin has been authorized in 24 countries, with over 80 million doses distributed worldwide. Pre-licensure clinical trials and post-marketing routine pharmacovigilance have yielded an extensive safety database, which attests to a favorable benefit-risk profile of Cecolin; notably, no signals indicative of an elevated risk of ADs have been identified to date [16,17,18]. While clinical trials represent a cornerstone of safety evaluation, their sample sizes are typically insufficient to detect rare adverse events. In contrast, post-marketing routine pharmacovigilance enables to collect adverse event reports at a population level, thus playing a crucial role in detecting unknown or rare adverse events possibly related to the vaccine. Nevertheless, such spontaneous reporting data are inherently constrained by several limitations, including under-reporting, the absence of denominator data, misclassification or miscoding issues, as well as biases stemming from the duration of market exposure and the prevailing reporting context [19]. Furthermore, although several systematic reviews have evaluated the safety of Cecolin and consistently supported its favorable safety profile, meta-analytic synthesis was not feasible for the outcome of ADs, owing to the limited data available [20,21].

Accordingly, to further elucidate the association between Cecolin vaccination and the incidence of ADs and thereby establish a more comprehensive understanding of the vaccine’s safety profile, we conducted this post-marketing real-world study. The primary objective was to compare the risk of ADs between females who received Cecolin and those who remained unvaccinated in China.

## 2. Materials and Methods

### 2.1. Data Sources and Study Population

Xiamen, a coastal city in southeast China, has over 5 million permanent residents. This post-marketing safety study utilized data from the Xiamen Health and Medical Big Data Center, which covers electronic health records for over 95% of the city’s residents and enables regional linkage of health information. Diagnostic, examination, laboratory, and prescription data were extracted from the electronic medical record system; vaccination records were obtained from the immunization information system; and maternal and neonatal information was sourced from the maternal and child health information system. Cross-system record linkage was accomplished through a unique citizen health identification number.

This study included females aged 9–45 years who (i) had at least two health-care records separated by ≥12 months in the Xiamen Health and Medical Big Data Center and (ii) had never received any HPV vaccine except Cecolin during the post-Cecolin period (1 September 2020–31 December 2023). Individuals with any pre-specified ADs diagnosed within one year before the index date were excluded. For each participant, the index date was defined as the latest of (i) 12 months after the first registration in the Xiamen Health and Medical Big Data Center; (ii) the 9th birthday; or (iii) 1 September 2020. Follow-up ended on the earliest of: (i) the 46th birthday; (ii) the first diagnosis date of study outcome; (iii) 31 December 2023; (iv) death; or (v) relocation to other cities.

The exposed cohort comprised participants who received at least one dose of Cecolin. The contemporaneously unexposed cohort consisted of females who had never received any HPV vaccine, matched in 1:4 to the exposed cohort based on calendar year and age. Sensitivity analyses evaluated incidence risks between the exposed cohort and a 1:4-matched historically unexposed cohort based on calendar year (minus 4 years) and age. For exposed participants, the index date was the date of their first Cecolin vaccination; for unexposed participants, the index date was the same date as that of their matched exposed counterpart (minus 4 years for the historical match). For both exposed and unexposed participants, follow-up ended on the earliest of: (i) 12 months after index date; (ii) the 46th birthday; (iii) the first diagnosis date of study outcome; (iv) 31 December 2023 (29 December 2019 for historically unexposed participants); (v) death; or (vi) relocation to other cities. This follow-up strategy was informed by previous HPV vaccine safety studies, and the 12-month follow-up period is consistent with recommendations for the assessment of AD risk in clinical trials of new vaccines [2,22].

In addition, this study has been registered at ClinicalTrials.gov on 24 January 2025 (NCT06824896).

### 2.2. New-Onset Autoimmune Diseases

Based on published studies [7,11,19,23,24] and a feasibility assessment aligned with the study objectives, we pre-specified ten ADs, including systemic lupus erythematosus (SLE), rheumatoid arthritis (RA), juvenile idiopathic arthritis (JIA), ankylosing spondylitis (AS), idiopathic thrombocytopenic purpura (ITP), Crohn’s disease, ulcerative colitis, type 1 diabetes, autoimmune thyroiditis, and optic neuritis (ON).

The final set of diseases retained for analysis would, however, be determined after case validation and evaluation of the identification algorithms.

All diagnostic records of each case were collated, and the dates of diagnosis were summarized. Patients without pre-specified ADs diagnosed within one year prior to the index date, but who received their first diagnosis of the specific ADs during the follow-up period, were defined as new-onset cases.

### 2.3. Case Validation and Identification Algorithms

Potential cases were first identified with an initial algorithm that required either the Chinese diagnostic terms or the corresponding International Classification of Diseases, 10th Revision (ICD-10) codes. For each disease, a set of validation samples was randomly extracted (10% by default; if <30 cases were extracted, the samples were increased to 30; if >200 cases were extracted, only 200 were kept; when the total number of algorithm-identified cases was <30, all of them were reviewed).

Two clinicians independently reviewed the extracted records and classified each candidate case as (i) fulfilling the case definition, (ii) unrelated diagnosis, or (iii) insufficient information for adjudication (missing imaging, laboratory data or prescriptions precluding judgment). Discrepancies were resolved by a third, senior clinician. Depending on the ADs under validation, the review panel comprised clinicians from rheumatology and clinical immunology, endocrinology, gastroenterology, and ophthalmology.

The positive predictive value (PPV) of the initial algorithm (by disease) was estimated from the case validation results. PPV was calculated as the percentage of confirmed cases meeting the case definition among all cases identified by the corresponding algorithm, with records classified as having insufficient information (category iii) excluded from the denominator. Algorithms with PPV > 70% were considered valid. For diseases with PPV ≤ 70%, the following refinements were added: (i) care setting (outpatient/emergency or inpatient), (ii) hospital level (tertiary, secondary and above, primary), and (iii) number of visits for the same disease (at least twice, at least three times). The algorithm yielding the highest PPV (>70%) was retained for final case identification; diseases whose PPV remained ≤70% after all refinements were considered unidentifiable and were excluded from the final analysis.

### 2.4. Statistical Analyses

To assess study feasibility, the person-years of follow-up required in the exposed group under a Poisson model for the comparison of incidence rates, assuming an exposed-to-unexposed ratio of 1:4, 80% power, a two-sided alpha of 0.05, different relative risks (2, 3, 5), and a range of background incidence rates for overall AD. Because all eligible individuals identified during the study period were enrolled, no fixed sample size was pre-specified; instead, the person-years actually accrued were compared against these requirements to ensure the study was adequately powered.

Demographic characteristics of the study population were summarized as counts, percentages, and means ± standard deviation (SD). Age-stratified incident cases and person-years were presented for each cohort, and the incidence rates (IRs) of ADs (per 100,000 person-years) and their 95% confidence intervals (CIs) were estimated. The outcome was characterized by an excessive proportion of zero counts, resulting in poor model fit and convergence issues under the standard Poisson regression model. Therefore, a zero-inflated Poisson model was employed to estimate incidence rate ratios (IRRs) with 95% CIs between cohorts, thereby better accommodating the observed data distribution by allowing for excess zeros. All statistical analyses were performed in R (version 4.3.0) using RStudio software (version 2023.12.0; Posit, PBC).

## 3. Results

### 3.1. Characteristics of the Participants

**Figure 1:**
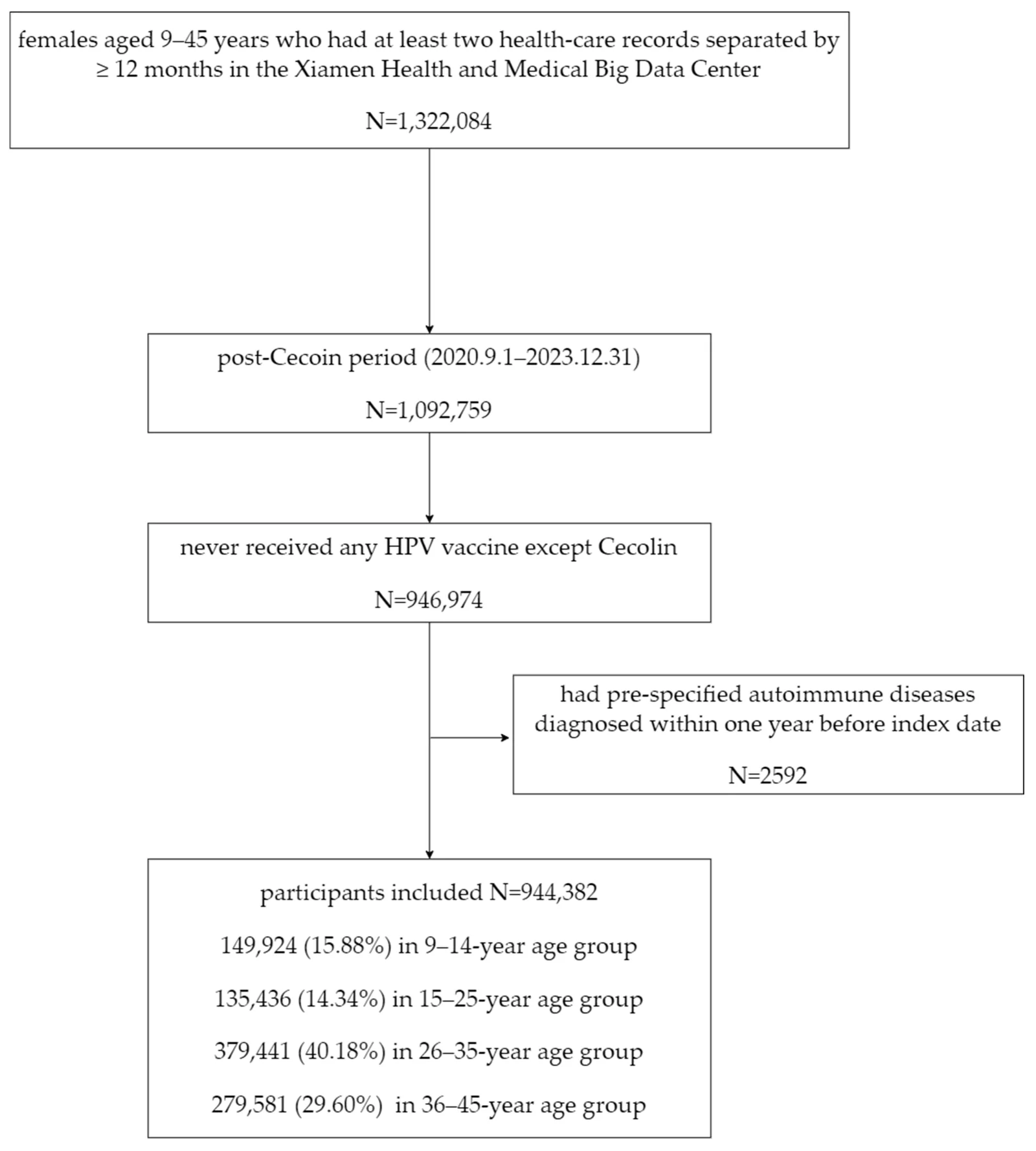
Flowchart of study population.

The study population selection flow is detailed in Figure 1. Overall, 944,382 females aged 9–45 years were included, accruing 2,871,317.0 person-years of follow-up. Mean age was 28.9 ± 10.48 years, the 26–35-year age group accounted for the largest proportion (40.18%), whereas the 15–25-year age group was the smallest (14.34%).

### 3.2. Performance of Identification Algorithms

PPVs of the case-identification algorithms for each disease are shown in Table 1. The initial algorithms (Chinese diagnostic terms or the corresponding ICD-10 codes) for SLE and ITP exceeded the 70% threshold (PPVs were 82.39% and 70.97% for SLE and ITP respectively); therefore, no refinement was applied. For JIA, RA, AS, and ON, the optimal algorithm was the initial algorithm plus at least three visits for the same disease. Algorithms for type 1 diabetes, autoimmune thyroiditis, Crohn’s disease and ulcerative colitis never achieved a PPV over 70% after any refinement and were excluded from the final analysis.

### 3.3. Surveillance of Autoimmune Diseases

As shown in Table 2, during the post-Cecolin period, 2028 incident cases of identifiable ADs were observed, yielding an overall incidence of 70.63 per 100,000 person-years (95% CI: 67.59–73.77). SLE was the most common disease with incidence of 29.08 per 100,000 person-years (95% CI: 27.14–31.12), while ON exhibited the lowest incidence (0.73 per 100,000 person-years, 95% CI: 0.45–1.12).

The overall incidence of identifiable ADs increased with age. A similar age gradient was observed for RA, rising from 0.81 per 100,000 person-years (95% CI: 0.17–2.37) in the 9–14-year group to 41.20 per 100,000 person-years (95% CI: 36.84–45.93) in the 36–45-year group. For AS, ON, ITP and SLE, incidence was lowest in the 9–14-year group and remained similar across the older age strata (15–25, 26–35 and 36–45-year group). JIA was detected exclusively in the 9–14-year group, consistent with its known predilection for children and adolescents under 16 years of age.

### 3.4. Associations Between Exposure to Cecolin and Autoimmune Diseases

A total of 92,026 participants in this study received at least one dose of Cecolin. Among them, 34,343 belonged to the 2006–2010 birth cohort (Table 3). Because an insufficient number of unvaccinated individuals was available in this birth cohort to support 1:4 matching, it was not included in the comparative analysis and was summarized descriptively only. All members of this cohort were aged 9–14 years; during follow-up, two ADs cases occurred (one JIA and one SLE), yielding an incidence of 1.76 per 100,000 person-years (95% CI: 0.21–6.37).

The remaining 57,683 exposed individuals constituted the matched exposed cohort, and each was matched with four unvaccinated females based on calendar year and age. The incidence of ADs was 41.20 per 100,000 person-years (95% CI: 28.18–58.17) in the matched exposed cohort and 52.55 per 100,000 person-years (95% CI: 44.79–61.27) in the contemporaneously unexposed cohort, yielding an IRR of 0.23 (95% CI: 0.08–0.65, *p* = 0.006). Disease-specific analyses indicated significant negative associations between the incidence of SLE and Cecolin vaccination (IRR = 0.21, 95% CI: 0.07–0.61, *p* = 0.004), and RA was similar (IRR = 0.20, 95% CI: 0.05–0.86, *p* = 0.031). No significant associations were observed for AS (IRR = 0.37, 95% CI: 0.12–1.17, *p* = 0.091) or ITP (IRR = 1.00, 95% CI: 0.99–1.00, *p* = 0.184). Regression analyses were not conducted for JIA or ON, as no cases were observed in either the exposed or unexposed cohorts.

Sensitivity analyses comparing the incidence risk between matched exposed cohort and historically unexposed cohort further confirmed the absence of an increased AD risk following Cecolin vaccination. Significant inverse associations were observed for SLE (IRR = 0.20, 95% CI: 0.06–0.62, *p* = 0.006), ITP (IRR = 0.37, 95% CI: 0.14–0.95, *p* = 0.039) and RA (IRR = 0.15, 95% CI: 0.03–0.77, *p* = 0.023), whereas no significant association was found for AS (IRR = 0.43, 95% CI: 0.15–1.19, *p* = 0.104).

## 4. Discussion

This large-scale longitudinal study estimated the incidence of ADs during the post-marketing period of Cecolin and found that the risk of developing ADs was either unrelated or significantly inversely associated with Cecolin vaccination.

To the best of our knowledge, no prior study in China has directly evaluated the risk of ADs after HPV vaccination, leaving a critical evidence gap that the present study was specifically designed to address. A systematic review estimated that the prevalence of ADs in China is 2.7–3.0%, with a female-to-male burden ratio of approximating 2:1 [25]. In the present study, the incidence of SLE was 29.08 per 100,000 person-years in the post-Cecolin period, close to the estimate of 26.41 per 100,000 person-years reported for urban Chinese females in 2017 [26]. ITP incidence was 4.35 per 100,000 person-years, slightly lower than the estimate of 7.59 per 100,000 observed among females in Yunnan province during 2022–2023, a difference possibly attributed to Yunnan’s higher altitude [27]. Published incidence rates of RA among Chinese females vary widely (17.8 to 160.1 per 100,000), this study’s estimate (22.15 per 100,000 person-years) fell within this ranges [28,29,30]. Our estimate is higher than the Global Burden of Disease (GBD) 2021 age-standardized incidence of RA reported for females globally (16.23 per 100,000) and in high-middle sociodemographic index (SDI) regions (16.21 per 100,000), but closely approximates the rate for females in high-SDI regions (24.27 per 100,000) [31]. This may be explained by the substantial differences in study design and data sources between the present population-based cohort and the GBD modeling approach, and by the fact that Xiamen is among the more economically developed regions in China. AS incidence in our study (15.78 per 100,000 person-years) exceeded the 1.30 to 5.71 per 100,000 person-years reported in Guangxi province in 2014–2021 (both sexes) [32]. Regrettably, no recent globally representative AS incidence data were identified; the available country-level female estimates—Thailand (11.7 per 100,000 person-years in 2020), South Korea (5.16 per 100,000 person-years in 2015), and the UK (3.3 per 100,000 person-years in 2017)—were all lower than our estimate [33,34,35]. Direct cross-study comparability, however, is limited by substantial differences in data sources, regions, study period and populations across these reports and the present cohort [32,33,34,35]. Incidence data for JIA and ON in China remain unclear; our findings were consistent with global estimates (JIA: 1.6 to 23 per 100,000 children; ON: 0.56 to 5.15 per 100,000) [36,37]. Overall, compared with previously reported epidemiological data, except for AS, the incidence of ADs observed in this study did not exceed the expected background level.

Most population-level observational studies did not support a causal association between AD and HPV vaccination [11]. For instance, a large linkage cohort study conducted in Denmark and Sweden demonstrated that HPV vaccination was not associated with an elevated risk of SLE, JIA, or ITP, with the relative risks (RRs) being 1.35 (95% CI: 0.69–2.67), 0.99 (95% CI: 0.78–1.26), and 1.18 (95% CI: 0.65–2.17), respectively [38]. Consistently, a large cohort study involving over 2 million girls in France also indicated that HPV vaccination did not lead to a significant increase in the incidence of cutaneous lupus erythematosus or SLE (adjusted hazard ratio [aHR] = 1.01, 95% CI: 0.69–1.48), RA or JIA (aHR = 0.97, 95% CI: 0.75–1.26), or ITP (aHR = 0.71, 95% CI: 0.48–1.08) [39]. Furthermore, multiple systematic reviews have synthesized evidence from diverse study types, including case reports, post-marketing surveillance data analyses, ecological studies, clinical trials, and population-level observational studies. Although several individual studies have suggested a potential positive correlation, the pooled evidence generally confirms that HPV vaccination does not elevate the risk of AD development [7,8,9,10,11]. For example, a case–control study reported an elevated risk of developing ADs following quadrivalent HPV vaccination, including SLE (odds ratio [OR] = 5.3, 95% CI: 1.5–20.5) [40]. Similarly, a register-based cohort study conducted in Denmark and Sweden identified an increased risk of certain ADs, such as Behçet’s syndrome, Raynaud’s disease, and type 1 diabetes, after quadrivalent HPV vaccination [38]. Conversely, a meta-analysis by Ferrari FA et al., which synthesized data from 14 controlled studies, revealed no significant increase in the overall risk of ADs following HPV vaccination (RR = 0.76, 95% CI: 0.55–1.03) [9]. In another systematic review, Willame C et al. integrated 22 post-licensure safety studies and indicated that HPV vaccination was not linked to an elevated risk of SLE, ITP, AS, JIA, RA, or ON, with all pooled estimates encompassing unity [10]. The discrepancy between those isolated positive findings and the consistent null results of pooled analyses most likely reflects methodological rather than biological factors: the individual studies were typically underpowered for detecting rare autoimmune events, increasing the probability that chance alone could yield a spurious positive association; they were also vulnerable to risks of bias arising from case or exposure ascertainment, inconsistent case definitions, and limited control for residual confounding [8,9,10,11].

In this study, comparisons between Cecolin-exposed and -unexposed cohorts showed a significant inverse association between ADs incidence and Cecolin vaccination in both contemporaneously matched (IRR = 0.23, 95% CI: 0.08–0.65; *p* = 0.006) and historically matched (IRR = 0.21, 95% CI: 0.07–0.66; *p* = 0.008) cohorts. Disease-specific analyses likewise showed either no association or a significant inverse association for each AD subtype studied. One important methodological caveat, however, warranted explicit acknowledgment when interpreting these findings—because the matching and adjustment in this study were restricted to calendar year and age, a range of established or suspected AD risk factors—including family history, smoking, alcohol use, socioeconomic status, drug exposure, etc.—were not matched on or adjusted for in the comparison, and the inverse association we observed should therefore be interpreted with caution rather than read as evidence of a true protective effect of vaccination [41,42]. Despite this residual confounding, the consistent direction and statistical significance of the association across both contemporaneously and historically matched comparisons, together with the lack of any positive signal in disease-specific analyses, provide reassuring evidence that Cecolin vaccination does not increase the risk of developing ADs. The observed negative association may be attributable to the following factors: first, during the study period, HPV vaccination program was provided exclusively to females aged 9–14 years, whereas older females accessed HPV vaccination voluntarily; thus, those who chose to be vaccinated might have greater health awareness and might be engaged in more preventive health behaviors, thereby reducing their baseline risk. Second, Cecolin might indirectly lower the likelihood of autoimmune risk by preventing HPV infection and associated chronic inflammation [43,44].

Population-based incidence data on ADs remain scarce in China. Leveraging city-wide medical big data, this study provides large-scale, longitudinal incidence estimates that help fill the evidence gap. Moreover, Cecolin was incorporated into China’s national immunization program for eligible females in October 2025; the real-world safety evidence generated here in Chinese population is directly relevant to programmatic deci-sion-making and to addressing public concerns about vaccine safety. Another strength is the case-identification process: rather than relying solely on pre-existing diagnostic codes, this study validated a sample of potential cases and iteratively refined the algorithms, thereby reducing misclassification bias.

Several limitations should be noted. Firstly, the 2006–2010 birth cohort contained a high proportion of vaccinated individuals, precluding 1:4 (or even 1:1) exposed-to-unexposed matching; consequently, this cohort was not included in comparative analyses between exposed and unexposed cohorts, which may limit inferences regarding associations between Cecolin vaccination and ADs. Secondly, based on the results of case validation, the data source adopted in the present study could not relatively accurately identify cases of type 1 diabetes, autoimmune thyroiditis and inflammatory bowel disease (Crohn’s disease and ulcerative colitis thyroiditis). Therefore, the association between Cecolin vaccination and the onset of these particular autoimmune diseases—conditions that warrant attention in HPV-vaccine safety surveillance—could not be reliably evaluated. Thirdly, the database was constructed for routine healthcare rather than research purposes; consequently, matching was restricted to calendar year and age. Although age represents an important determinant of AD incidence, and calendar year may partially balance certain environmental exposures between groups (e.g., climate, air pollution, and ultraviolet radiation), bias or confounding arising from unmeasured health-related factors could not be avoided. In particular, we did not capture socio-demographic and lifestyle factors that are known or suspected to interact with genetic predisposition in the induction of ADs [41,42]; the potential confounding or effect-modifying roles of these gene–environment interactions in the observed associations could therefore not be assessed in the present analysis. Furthermore, the following inherent constraints of administrative data exist: (i) out-of-city medical or HPV vaccination records were not captured due to population mobility; and (ii) the study population was limited to individuals registered in the Xiamen Health and Medical Big Data Center, restricting generalizability and warranting caution when extrapolating the findings.

## 5. Conclusions

In summary, this study represents the first real-world evaluation of the autoimmune safety profile of Cecolin, a Chinese bivalent HPV vaccine, using population-based cohort data. Consistent with prior evidence, our findings further confirm that Cecolin vaccination does not increase the risk of ADs, thereby contributing robust real-world evidence that supports its post-marketing safety profile. These results may inform vaccination policy, enhance public confidence, and guide post-licensure safety monitoring in China and other countries that have introduced or are considering introducing this bivalent HPV vaccine into their national immunization programs.

Future studies should extend these findings through large-scale prospective cohorts with linkage to detailed socio-demographic and environmental data. Continued post-marketing active surveillance—integrating immunization registries, electronic health records, and patient-reported outcomes—remains essential, particularly following Cecolin’s incorporation into China’s national immunization program in October 2025.

## Figures and Tables

**Table 1:** Positive predictive values (PPVs) of case-identification algorithms.

Autoimmune Diseases	Initial Algorithm ^a^	Initial Algorithm Plus Following Refinements						
		(i) Care Setting		(ii) Hospital Level			(iii) Number of Visits for the Same Disease	
		Inpatient (95% CI ^b^)	Outpatient/Emergency (95% CI ^b^)	Tertiary (95% CI ^b^)	Secondary and Above (95% CI ^b^)	Primary (95% CI ^b^)	At Least Twice (95% CI ^b^)	At Least Three Times (95% CI ^b^)
SLE	82.39 (76.13, 88.66)	NA ^c^	NA ^c^	NA ^c^	NA ^c^	NA ^c^	NA ^c^	NA ^c^
ITP	70.97 (54.99, 86.95)	NA ^c^	NA ^c^	NA ^c^	NA ^c^	NA ^c^	NA ^c^	NA ^c^
JIA	37.93 (20.27, 55.59)	60.00 (35.21, 84.79)	14.29 (0.00, 32.62)	37.93 (20.27, 55.59)	37.93 (20.27, 55.59)	- ^d^	77.78 (50.62, 100.00)	85.71 (59.79, 100.00)
RA	35.63 (28.20, 43.05)	55.56 (23.09, 88.02)	34.44 (26.86, 42.02)	39.42 (31.23, 47.60)	39.42 (31.23, 47.60)	13.04 (0.00, 26.81)	78.33 (67.91, 88.76)	84.00 (73.84, 94.16)
AS	60.58 (51.18, 69.97)	68.75 (46.04, 91.46)	59.09 (48.82, 69.36)	62.00 (52.49, 71.51)	62.00 (52.49, 71.51)	25.00 (0.00, 67.44)	63.79 (51.42, 76.16)	77.08 (65.19, 88.97)
ON	30.77 (5.68, 55.86)	25.00 (0.00, 67.44)	33.33 (2.53, 64.13)	30.77 (5.68, 55.86)	30.77 (5.68, 55.86)	- ^d^	60.00 (17.06, 100.00)	100.00 (-)
Type 1 diabetes	6.67 (0.00, 15.59)	50.00 (0.00, 100.00)	3.57 (0.00, 10.45)	5.88 (0.00, 17.07)	5.88 (0.00, 17.07)	7.69 (0.00, 22.18)	25.00 (0.00, 55.01)	25.00 (0.00, 55.01)
Autoimmune thyroiditis	4.95 (0.72, 9.18)	0.00 (-)	5.88 (0.88, 10.88)	5.21 (0.76, 9.65)	5.10 (0.75, 9.46)	0.00 (-)	9.09 (0.60, 17.59)	9.09 (0.60, 17.59)
Crohn’s disease	44.00 (24.54, 63.46)	66.67 (35.87, 97.47)	31.25 (8.54, 53.96)	44.00 (24.54, 63.46)	44.00 (24.54, 63.46)	- ^d^	58.33 (30.44, 86.23)	63.64 (35.21, 92.06)
Ulcerative colitis	5.33 (0.25, 10.42)	13.04 (0.00, 26.81)	1.92 (0.00, 5.66)	13.04 (0.00, 26.81)	13.04 (0.00, 26.81)	1.92 (0.00, 5.66)	13.04 (0.00, 26.81)	13.04 (0.00, 26.81)

SLE, systemic lupus erythematosus; ITP, idiopathic thrombocytopenic purpura; JIA, juvenile idiopathic arthritis; RA, rheumatoid arthritis; AS, ankylosing spondylitis; ON, optic neuritis. ^a^ Initial algorithm was defined as Chinese diagnostic terms or the corresponding ICD-10 codes. ^b^ CI, confidence interval, calculated by the Clopper–Pearson exact method. ^c^ NA, not applicable, SLE and ITP already exceeded the 70% threshold with the initial algorithm; therefore, no refinement was applied. ^d^ When an algorithm identified zero cases that fulfilled the case definition, the PPV could not be derived and was indicated by “-”.

**Table 2:** Incidence of identifiable autoimmune diseases in post-Cecolin period.

Identifiable Autoimmune Diseases ^a^ (Age Group)	Post-Cecolin Period (1 September 2020–31 December 2023)	
	n ^b^ (N_Y_^c^)	IR ^d^ (95% CI ^e^)
Overall incidence of identifiable autoimmune diseases		
9–14-year	93 (370,310.5)	25.11 (20.27, 30.77)
15–25-year	273 (450,489.7)	60.60 (53.62, 68.23)
26–35-year	922 (1,261,696.5)	73.08 (68.43, 77.95)
36–45-year	740 (788,820.3)	93.81 (87.17, 100.82)
9–45-year	2028 (2,871,317.0)	70.63 (67.59, 73.77)
Incidence by category		
Systemic lupus erythematosus		
9–14-year	48 (370,310.5)	12.96 (9.56, 17.19)
15–25-year	155 (450,489.7)	34.41 (29.20, 40.27)
26–35-year	388 (1,261,696.5)	30.75 (27.77, 33.97)
36–45-year	244 (788,820.3)	30.93 (27.17, 35.07)
9–45-year	835 (2,871,317.0)	29.08 (27.14, 31.12)
Idiopathic thrombocytopenic purpura		
9–14-year	13 (370,310.5)	3.51 (1.87, 6.00)
15–25-year	19 (450,489.7)	4.22 (2.54, 6.59)
26–35-year	60 (1,261,696.5)	4.76 (3.63, 6.12)
36–45-year	33 (788,820.3)	4.18 (2.88, 5.88)
9–45-year	125 (2,871,317.0)	4.35 (3.62, 5.19)
Juvenile idiopathic arthritis		
9–14-year	22 (370,310.5)	5.94 (3.72, 8.99)
15–25-year	0 (450,489.7)	0.00 (0.00, 0.82)
26–35-year	0 (1,261,696.5)	0.00 (0.00, 0.29)
36–45-year	0 (788,820.3)	0.00 (0.00, 0.47)
9–45-year	22 (2,871,317.0)	0.77 (0.48, 1.16)
Rheumatoid arthritis		
9–14-year	3 (370,310.5)	0.81 (0.17, 2.37)
15–25-year	47 (450,489.7)	10.43 (7.67, 13.87)
26–35-year	261 (1,261,696.5)	20.69 (18.25, 23.35)
36–45-year	325 (788,820.3)	41.20 (36.84, 45.93)
9–45-year	636 (2,871,317.0)	22.15 (20.46, 23.94)
Ankylosing spondylitis		
9–14-year	11 (370,310.5)	2.97 (1.48, 5.32)
15–25-year	54 (450,489.7)	11.99 (9.00, 15.64)
26–35-year	240 (1,261,696.5)	19.02 (16.69, 21.59)
36–45-year	148 (788,820.3)	18.76 (15.86, 22.04)
9–45-year	453 (2,871,317.0)	15.78 (14.36, 17.30)
Optic neuritis		
9–14-year	1 (370,310.5)	0.27 (0.01, 1.50)
15–25-year	4 (450,489.7)	0.89 (0.24, 2.27)
26–35-year	8 (1,261,696.5)	0.63 (0.27, 1.25)
36–45-year	8 (788,820.3)	1.01 (0.44, 2.00)
9–45-year	21 (2,871,317.0)	0.73 (0.45, 1.12)

^a^ According to the results of case validation and identification algorithms, identifiable autoimmune diseases in this study included systemic lupus erythematosus, idiopathic thrombocytopenic purpura, juvenile idiopathic arthritis, rheumatoid arthritis, ankylosing spondylitis and optic neuritis. ^b^ Number of cases. ^c^ N_Y_, person-years for all participants in each group. ^d^ IR, incidence rate, here means incidence density (per 100,000 person-years). ^e^ CI, confidence interval, estimated by exact Poisson methods.

**Table 3:** Incidence and comparisons between exposed and unexposed cohorts.

Identifiable ADs (9–45-Year Age Group)	Exposed Cohorts N ^a^ = 92,026				Unexposed Cohorts							
	Unmatched Exposed Cohort (Belonged to the 2006–2010 Birth Cohort) N ^a^ = 34,343		Matched Exposed Cohort N ^a^ = 57,683		Contemporaneously Unexposed Cohort N ^a^ = 230,732				Historically Unexposed Cohort N ^a^ = 230,732			
	n ^b^ (N_Y_ ^c^)	IR ^d^ (95% CI ^e^)	n ^b^ (N_Y_ ^c^)	IR ^d^ (95% CI ^e^)	n ^b^ (N_Y_ ^c^)	IR ^d^ (95% CI ^e^)	IRR ^f^ (95% CI ^g^)	*p* Value	n ^b^ (N_Y_ ^c^)	IR ^d^ (95% CI ^e^)	IRR ^f^ (95% CI ^g^)	*p* Value
Overall incidence	2 (113,375.3)	1.76 (0.21, 6.37)	32 (77,663.9)	41.20 (28.18, 58.17)	163 (310,169.0)	52.55 (44.79, 61.27)	0.23 (0.08, 0.65)	0.006	189 (308,872.6)	61.19 (52.78, 70.56)	0.21 (0.07, 0.66)	0.008
Incidence by category												
SLE	1 (113,375.3)	0.88 (0.02, 4.91)	12 (77,663.9)	15.45 (7.98, 26.99)	69 (310,169.0)	22.25 (17.31, 28.15)	0.21 (0.07, 0.61)	0.004	76 (308,872.6)	24.61 (19.39, 30.80)	0.20 (0.06, 0.62)	0.006
ITP	0(113,375.3)	0.00 (0.00, 3.25)	2 (77,663.9)	2.58 (0.31, 9.30)	9 (310,169.0)	2.90 (1.33, 5.51)	1.00 (0.99, 1.00)	0.184	14 (308,872.6)	4.53 (2.48, 7.60)	0.37 (0.14, 0.95)	0.039
JIA	1 (113,375.3)	0.88 (0.02, 4.91)	0 (77,663.9)	0.00 (0.00, 4.75)	0 (310,169.0)	0.00 (0.00, 1.19)	-	-	0 (308,872.6)	0.00 (0.00, 1.19)	-	-
RA	0(113,375.3)	0.00 (0.00, 3.25)	8 (77,663.9)	10.30 (4.45, 20.30)	52 (310,169.0)	16.77 (12.52, 21.99)	0.20 (0.05, 0.86)	0.031	76 (308,872.6)	24.61 (19.39, 30.80)	0.15 (0.03, 0.77)	0.023
AS	0(113,375.3)	0.00 (0.00, 3.25)	11 (77,663.9)	14.16 (7.07, 25.34)	33 (310,169.0)	10.64 (7.32, 14.94)	0.37 (0.12, 1.17)	0.091	28 (308,872.6)	9.07 (6.02, 13.10)	0.43 (0.15, 1.19)	0.104
ON	0(113,375.3)	0.00 (0.00, 3.25)	0 (77,663.9)	0.00 (0.00, 4.75)	1 (310,169.0)	0.32 (0.01, 1.80)	-	-	0 (308,872.6)	0.00 (0.00, 1.19)	-	-

ADs, autoimmune diseases; SLE, systemic lupus erythematosus; ITP, idiopathic thrombocytopenic purpura; JIA, juvenile idiopathic arthritis; RA, rheumatoid arthritis; AS, ankylosing spondylitis; ON, optic neuritis. ^a^ Number of participants included in each cohort. ^b^ Number of cases. ^c^ N_Y_, person-years for all participants in each group. ^d^ IR, incidence rate, here means incidence density (per 100,000 person-years). ^e^ CI, confidence interval, estimated by exact Poisson methods. ^f^ IRR, incidence rate ratio, the displayed values were matched exposed cohort vs. contemporaneously/historically unexposed cohort. ^g^ Estimated by Wald methods.

## Data Availability

All data of this study are accessible in the Xiamen Health and Medical Big Data Center to authorized researchers, institutional review board, and regulatory authorities. Aggregated, de-identified data analyzed in this study are available from the corresponding authors upon reasonable request.

## Abbreviations

The following abbreviations are used in this manuscript:

AD	Autoimmune Disease
aHR	Adjusted Hazard Ratio
AS	Ankylosing Spondylitis
CI	Confidence Interval
GBD	Global Burden of Disease
HPV	Human Papillomavirus
ICD-10	International Classification of Diseases, 10th Revision
IR	Incidence Rate
IRR	Incidence Rate Ratio
ITP	Idiopathic Thrombocytopenic Purpura
JIA	Juvenile Idiopathic Arthritis
NA	Not Applicable
ON	Optic Neuritis
OR	Odds Ratio
PPV	Positive Predictive Value
RA	Rheumatoid Arthritis
RR	Relative Risk
SD	Standard Deviation
SDI	Sociodemographic Index
SLE	Systemic Lupus Erythematosus
WHO	World Health Organization
